# Hyperthyroidism With Non-chylous Ascites: A Case Report

**DOI:** 10.7759/cureus.46657

**Published:** 2023-10-07

**Authors:** Shodai Takahashi, Kasumi Satoh, Manabu Okuyama, Nobuhisa Hirasawa, Hajime Nakae

**Affiliations:** 1 Department of Hematology, Nephrology, and Rheumatology, Akita University Graduate School of Medicine, Akita, JPN; 2 Department of Emergency and Critical Care Medicine, Akita University Graduate School of Medicine, Akita, JPN

**Keywords:** non-chylous ascites, rapid turnover protein, graves's disease, emergency department, hyperthyroidism, ascites

## Abstract

Ascites is the accumulation of fluid in the abdominal cavity and is commonly attributed to various etiologies, including portal hypertension and peritoneal diseases. Hyperthyroidism is rarely associated with ascites, which is typically chylous and accompanied by high central venous pressure. We present a unique case of a 57-year-old woman with untreated hyperthyroidism who manifested non-chylous ascites without evidence of high venous pressure. Initially presenting with left lower leg pain, the patient presented with leg edema, abdominal distention, and diarrhea. A range of diagnostic tests ruled out common etiologies of ascites, such as liver cirrhosis, renal impairment, heart failure, infection, and malignancy. Ascites was characterized by low triglyceride levels, while no evidence of high venous pressure was found. Notably, the patient showed decreased levels of rapid turnover proteins, suggesting hypercatabolism and insufficient protein synthesis due to hyperthyroidism. Upon the initiation of antithyroid therapy, the patient’s symptoms markedly improved. In conclusion, this report highlights a rare manifestation of hyperthyroidism that resulted in non-chylous ascites without high venous pressure. This underscores the need to include hyperthyroidism in the differential diagnosis of unexplained ascites, particularly in cases in which classical hyperthyroid symptoms are absent.

## Introduction

Fluid accumulation in the abdominal cavity results in ascites. The underlying etiologies of ascites include portal hypertension, hypoproteinemia, peritoneal diseases, myxedema, and trauma [1,2].

The ascites associated with hyperthyroidism have been reported in several cases, although it is not a typical symptom [3-5]. Some cases of thyroid diseases do not present with classic symptoms and signs [6-8]; therefore, thyroid disease should be considered a case of ascites to avoid delays in treatment.

The etiology of hyperthyroidism-related ascites reported previously was explained by high central venous pressure, and the characteristic fluid was chylous ascites [3-5]. High central venous pressure increases augmented capillary filtration and impedes lymphatic drainage, resulting in ascites [3-5]. Chylous ascites is characterized by milky ascitic fluid with a high triglyceride concentration. Although the literature varies, chylous ascites is often defined as triglyceride levels >200 mg in ascites [9-11].

This report describes a case of hyperthyroidism with non-chylous ascites without the manifestation of high venous pressure.

## Case presentation

A 57-year-old woman with pain in the left lower leg was transported to our emergency department. She also complained of leg edema, abdominal distention, and diarrhea for the past three months. Five years prior, she was diagnosed with hyperthyroidism at another hospital; however, she did not receive any treatment thereafter by will. The patient had no history of smoking or alcohol consumption. Apart from hyperthyroidism, her medical history was unremarkable. She denied any history of abdominal trauma, surgery, or pelvic irradiation.

Her vital signs were as follows: blood pressure of 161/87 mmHg; pulse rate of 140/min with regular rhythm; body temperature of 37.8 °C; oxygen saturation of 98% in ambient air. On physical examination, the upper body was markedly thin; in contrast, her abdomen was distended, and pitting edema was obvious in her lower body. Point-of-care ultrasonography revealed the presence of a large amount of ascites and no distended inferior vena cava. Chest radiography revealed a cardiothoracic ratio of 53%, a dull costophrenic angle, and no opacities. Electrocardiography revealed no irregular rhythms, ST segment changes, heart blocks, or bundle branch blocks. The heart rate was 131 beats per minute with sinus rhythm.

The laboratory findings on admission are shown in Table 1.

**Table 1:** Laboratory findings

Investigations	Value	Reference range levels
Blood count test		
White blood cell count	8,300	3,300 - 8,600 /μL
Hemoglobin	12.4	11.6 - 14.8 g/dL
Platelet count	336	158 - 348 10^3^/μL
Coagulation test		
Prothrombin time-international normalized ratio	1.15	1.10 - 0.90
Prothrombin time (percentage activity)	80.6	80 - 120 %
Activated partial thromboplastin time	25.7	25.0 - 32.0 s
Serum biochemistry test		
Aspartate aminotransferase	41	13 - 30 U/L
Alanine transaminase	22	7 - 23 U/L
Alkaline phosphatase	201	38 - 113 U/L
Lactate dehydrogenase	350	124 - 222 U/L
Gamma-glutamyl transferase	12	9 - 32 U/L
Total bilirubin	0.3	0.4 - 1.5 mg/dL
Creatinine kinase	272	41 - 153 U/L
Total protein	4.2	6.6 - 8.1 g/dL
Albumin	1.4	4.1 - 5.1 g/dL
Blood urea nitrogen	8.5	8.0 - 20.0 mg/dL
Creatinine	0.39	0.46 - 0.79 mg/dL
Uric acid	2.7	7.0 - 2.3 mg/dL
Calcium (albumin correction)	8.8	8.8 - 10.1 mg/dL
Sodium	140	138 - 145 mEq/L
Potassium	2.8	3.6 - 4.8 mEq/L
Chloride	105	101 - 108 mEq/L
Iron	66	40 - 188 μg/dL
Total iron-binding capacity	166	246 - 410 μg/dL
Unsaturated iron-binding capacity	100	180 - 270 μg/dL
Ferritin	159.3	12 - 60 ng/mL
C-reactive protein	0.04	0 - 0.14 mg/dL
Glucose	130	100 - 140 mg/dL
Brain natriuretic peptide	58.2	0 - 18.4 pg/mL
Thyroid related test		
Free triiodothyronine (T3)	13.5	2.3 - 4.0 pg/mL
Free thyroxine (T4)	4.27	0.9 - 1.7 pg/mL
Thyroid-stimulating hormone (TSH)	0.005	0.5 - 5.0 μIU/mL
Thyroglobulin	21.9	0 - 35.1 ng/mL
Thyroglobulin antibody	491	0 - 19.3 IU/mL
Thyroid peroxidase antibody	193	0 - 3.3 IU/mL
TSH receptor antibody	11.1	0 - 2.0 IU/L
Rapid turnover protein test		
Transthyretin	9	22 - 34 mg/dL
Retinol-binding protein	1.0	1.9 - 4.6 mg/dL
Transferrin	138	190 - 320 mg/dL

**Figure 1:**
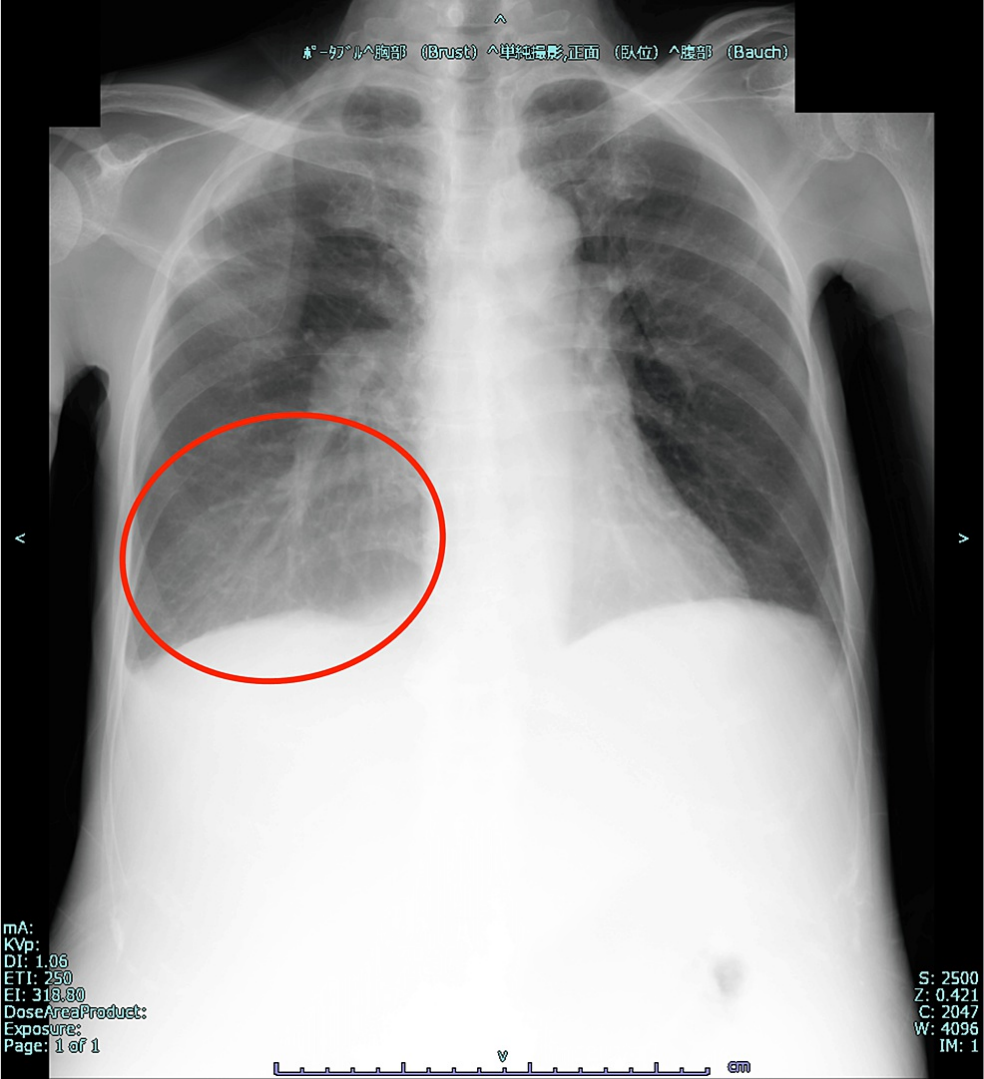
Chest radiograph (prone) Right pleural effusion (red round) and no evidence of pulmonary congestion or cardiac enlargement are observed.

Blood count and coagulation test results were grossly normal, and biochemical tests did not indicate liver cirrhosis or renal impairment. A mild elevation of creatine kinase levels and obvious hypoalbuminemia and hypokalemia were noted. No obvious pathological elevation of brain natriuretic peptide (BNP) was observed. Therefore, the patient’s heart failure test results were negative. Thyroid-related tests revealed obvious hyperthyroidism, suggestive of Graves' disease. Thyroid ultrasonography revealed a diffusely enlarged thyroid gland and increased blood flow. Levels of rapid turnover proteins such as transthyretin (TTR), retinol-binding protein (RBP), and transferrin, measured at a later date, were low. Chest radiography revealed a right pleural effusion with no evidence of pulmonary congestion or cardiac enlargement (Figure 1).

**Figure 2:**
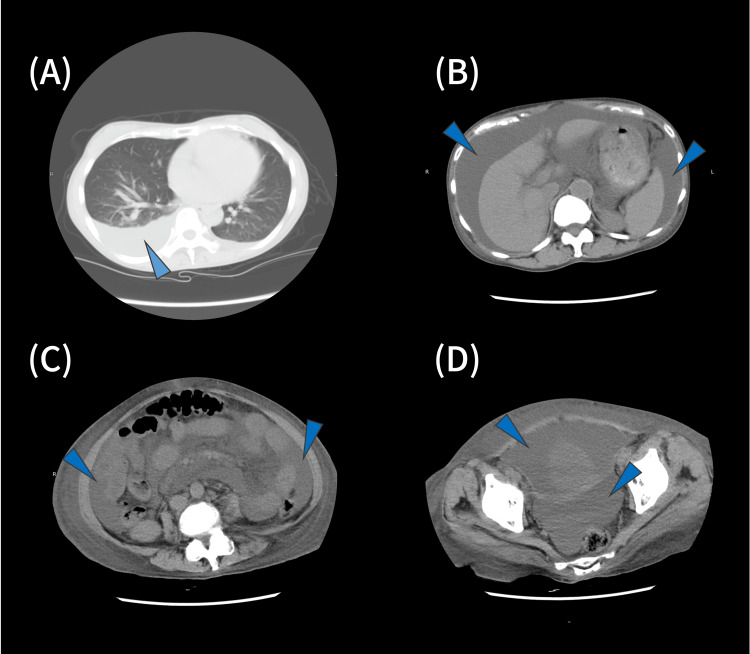
A non-enhanced computed tomography (chest to pelvis) (A) chest imaging; (B) abdominal imaging (upper); (C) abdominal imaging (middle); (D) abdominal imaging (lower). Right pleural effusion and ascites (blue arrowhead) were observed, but no obvious malignancy was detected.

Non-enhanced computed tomography revealed right pleural effusion and ascites (Figure 2); however, no obvious malignancy was observed.

The ultrasound showed thyroid enlargement and increased intrathyroidal blood flow, known as the thyroid inferno sign. An abdominocentesis was performed, and fluid analysis revealed non-chylous ascites (triglyceride level: 75 mg/dL, total protein level: <0.5 g/dL, lactate dehydrogenase level: 41 IU/L, albumin level: 0.2 g/dL). The results of the microbiological culture and cytological analysis of the ascites were negative. Therefore, infectious and malignant ascites were ruled out.

The patient was diagnosed with hyperthyroidism, hypoproteinemia, and hypopotassemia. Her chief complaint was left lower leg pain, which was explained by muscle spasms due to hypopotassemia.

Further examinations and treatment were deemed necessary after admission. Treatment with thiamazole (15 mg/day) and potassium iodine (50 mg/day) was initiated for hyperthyroidism. Her signs and symptoms of edema, diarrhea, hypopotassemia, ascites, and pleural effusion gradually improved after intravenous albumin and oral potassium replacement. However, hypoproteinemia persisted. She also received landiolol hydrochloride for heart rate regulation, and her tachycardia gradually improved.

The patient’s free triiodothyronine (T3) and thyroxine (T4) levels improved and almost normalized by day 10. She was discharged on day 20 and continued to receive antithyroid drugs in an outpatient setting. No ascites, edema, or diarrhea recurrence occurred after discharge, and her free T3 and T4 levels remained within the normal range.

## Discussion

Here, we report a case of non-chylous ascites as a manifestation of hyperthyroidism without evidence of high venous pressure. Levels of rapid turnover proteins decreased, suggesting hypercatabolism and protein synthesis insufficiency due to hyperthyroidism. The patient’s subjective symptoms improved after receiving antithyroid therapy.

Hyperthyroidism-induced ascites has been described in a few previous case reports as chylous ascites [3-5]. In these cases, triglyceride levels in the ascitic fluid were 132-347 mg/dL. In contrast, the ascites triglyceride level in the present case was 75 mg/dL, which was defined as non-chylous ascites. The lesson learned from the current case is that ascites is a possible symptom of hyperthyroidism, and its features can be non-chylous. In our case, based on the self-interruption of treatment for hyperthyroidism, the patient was promptly suspected to have a correct diagnosis. However, some cases of thyroid disease may be difficult to diagnose because of the absence of classic symptoms and signs. Boelaert reported that the classic symptoms and signs of hyperthyroidism are significantly less common, especially in the elderly, and recommended a lower threshold for performing thyroid function tests [7]. The inclusion of hyperthyroidism in the differential diagnosis of unexplained ascites may prevent delays in diagnosis and treatment.

In the previous three case reports of ascites as a symptom of hyperthyroidism, the authors suggested the mechanisms of chylous ascites in hyperthyroidism due to central venous hypertension. Hiroi et al. have reported a case of chylous ascites and chylothorax accompanied by hyperthyroidism. The authors concluded that chylous ascites and chylothorax were manifestations of secondary to lymphatic hypertension [3]. Hsieh and Uchihara also concluded that heart failure due to hyperthyroidism is a cause of chylous ascites [4,5]. High central venous pressure increases abdominal lymph production due to augmented capillary filtration, impedes lymphatic drainage, and decreases effective collateral flow. However, the novelty of our case is that the ascites were not non-chylous. Furthermore, increased venous pressure was not observed. Ultrasonography revealed no dilatation of the inferior vena cava, and chest radiography did not show signs of congestion. Farias et al. reported that serum BNP levels discriminate heart failure-related ascites from other causes. A cutoff of >364 pg/mL (sensitivity, 98%; specificity, 99%) had the highest positive likelihood ratio (168.1), and a cutoff of ≤182 pg/mL had the lowest negative likelihood ratio (0.0) [12]. In our case, the serum BNP level was 58.2 pg/mL, suggesting that heart failure was not the cause of the ascites. This implies that the ascites in our case were not due to heart failure or high central venous pressure, but other etiologies causing ascites with hyperthyroidism were considered. Generally, a serum albumin ascites gradient (SAAG) of >1.1 g/dL suggests portal hypertension-related ascites and the SAAG was 1.1 in our case. In this case, SAAG was at the cutoff value, making it unhelpful for differential diagnosis. Furthermore, our patient did not have any clinical features of cirrhosis that ruled out portal hypertension-related ascites. Levels of rapid turnover proteins, such as TTR, RBP, and transferrin, were decreased in our patient. This suggests that hypoproteinemia due to hypermetabolism and inadequate protein biosynthesis caused by hyperthyroidism contribute to ascites. This point of view is unique to this case and requires further investigation.

## Conclusions

Here, we report the first case of non-chylous ascites as a manifestation of hyperthyroidism. Ascites complicated by hyperthyroidism is rare. However, as the absence of classical symptoms and signs of hyperthyroidism often result in delayed diagnosis and treatment, ascites could be a significant sign of hyperthyroidism. In the present case, no signs of high venous pressure were observed. Levels of rapid turnover proteins were low, implying hypercatabolism and insufficient protein synthesis in hyperthyroidism. This suggests that ascites is associated with hyperthyroidism.
